# Intercorporeal collaboration: Staging, parsing, and embodied directives in dementia care

**DOI:** 10.1177/13634593231173809

**Published:** 2023-05-22

**Authors:** Lars-Christer Hydén, Anna Ekström, Ali Reza Majlesi

**Affiliations:** Linköping University, Sweden; Stockholm University, Sweden

**Keywords:** discourse and conversation analysis, environment and health, chronic illness and disability

## Abstract

This study shows how concerted bodily movements and particularly intercorporeality play a central role in interaction, particularly in joint activities with people with late-stage dementia. Direct involvement of bodies in care situations makes intercorporeal collaboration the basic form for engaging with people with late-stage dementia. By detailed analysis of a videorecording of a joint activity involving a person with late-stage dementia as an example, we show that the process of concerted bodily movements includes not only an interactive bodywork but also a reconfiguration of the routine activities and actions in situ. Reconfigurations often require, and are the outcome of, particular practices for the systematic modification of the embodied conducts of the participants and their use of artifacts in the surrounding environment. These practices, that we highlight in our study, are (1) staging activities through organization and re-organization of body parts, as well as artifacts (rather than using verbal descriptions of activities); (2) decomposing (parsing) activities into smaller parts possible for the person with dementia to perform (rather than using verbal action descriptions); and (3) providing embodied directions and bodily demonstrations of actions (rather than using verbal directives). As a result, we point to these practices for their reflexive roles in the change of the use of modalities in interaction: from mainly using verbal language to the prominence of visual depiction and bodily demonstration as necessary methods to facilitate the participation of people with latestage dementia in joint activities

## Introduction

Intercorporeal collaboration can be defined as *concerted bodily movements* constituting the main media for conducting an activity rather than exchange of spoken language. It means that bodily movements are seen and treated as identifiable and expressive, and thus as meaningful *actions* (cf. Meyer and Wedelstaedt, 2017). Typical examples of activities that are based on intercorporeal interaction and collaboration would be craft-work instructional activities (e.g. Ekström and Lindwall, 2014), dance (e.g. Keevallik and Ekström, 2019), and sport activities (e.g. Råman, 2019). In a similar fashion, many intimate activities performed in close interpersonal relations take place mainly between bodies (cf. Goodwin and Cekaite, 2018; Hydén et al., 2022; Meyer and Wedelstaedt, 2017; Meyer et al., 2017).

As a point of departure, we consider “caring” as one social domain where intercorporeal collaboration tends to be the basic form for engaging in joint activities. This is true both about care in the everyday sphere—for example, taking care of young children at home (Goodwin and Cekaite, 2018)—as well as in the professional sphere, not the least in elder care. In the context of professional caregiving, intercorporeal collaboration often means physically supporting the patient or client in such a way that it becomes possible for that person to move, modifying their bodily position, and taking part in activities, for instance in physiotherapy (Martin and Sahlstrom, 2010) or dementia care (Hydén et al., 2022). In these activities, collaboration between the therapist or nurse and the patient or client involves the participants’ active and lived bodies. The body is not only a resource or a site for implementing practices but there is an interactive relation between bodies, something that is part and parcel of *interactive bodywork* previously noticed in professional practices (cf. Twigg, 2000; see also Heath, 1986, 2006). In such activities, the bodies in collaboration are what Merleau-Ponty called *expressive bodies* that are constitutive of ongoing actions (Merleau-Ponty, 2014, chapter 6).

For this study, we use an example of a caregiving activity involving persons living with late-stage dementia as a case in point. For people living with late-stage dementia the lifeworld shrinks and becomes increasingly centered around the body and its needs. Participating in language-based activities like a quiz or storytelling activities is no longer easily possible. Further, persons living with late-stage dementia need support to perform basic, habitual routine activities and actions like eating and walking, which they previously could perform on their own. Thus, individually performed routine actions are turned into supported joint actions (Hydén et al., 2022). As the use of spoken language is severely constrained, in supportive work with people living with late-stage dementia, intercorporeal collaboration to perform routine care activities becomes more prominent. This is a situation similar to caregiving involving people with other kinds of severe communicative and cognitive disabilities where spoken language can no longer function as the main organizational and communicative media (cf. for instance, Goode, 1990). Despite the ubiquity of interactive bodywork and thus intercorporeal collaboration in everyday and professional settings, empirical research on the organization and performance of collaboration and interaction when it is neither based on, nor organized by, linguistic resources, is still limited (but see, Goodwin and Cekaite, 2018; Meyer et al., 2017).

With a focus on care with people living with late-stage dementia, what is of interest to us is how intercorporeal collaboration is organized and how concerted bodily routine actions are produced and coordinated without the use of spoken language as the primary method for communicating and making actions. We specifically intend to investigate how participants reconfigure routine activities and actions in order for a person living with late-stage dementia to participate in those activities. As a case in point, we use a videorecording of an activity where a person living with late-stage dementia, who is still able to stand for a short while and utter at least some words, is assisted to be engaged in peeling and scrubbing potatoes as part of the preparation of dinner at the residential nursing home where she lives.

## Intercorporeal supportive guidance

People living with late-stage dementia, in many respects of their lives, are severely challenged as they are constrained in terms of using cognitive, linguistic, and motor abilities (Astell and Ellis, 2004; Boller et al., 2002; Ellis and Astell, 2010). In most cases, it is no longer possible for the individual to perform routine actions or combination of actions (Giovannetti et al., 2002). The expressive functions of individuals in later-stages are reduced to gaze movements, restricted bodily motions, and some basic forms of verbal vocalizations. Unfortunately, the research about people living with late-stage dementia as participants in various activities is limited. A few studies have looked at basic activities among people with moderate to advanced dementia like eating and mealtime activities (Athlin and Norberg, 1987; Hydén et al., 2022; Sandman et al., 1988), assisted moving and sitting down (Majlesi et al., 2021); and some studies have looked at participation in more complex activities like knitting (Boller et al., 2002), making music (Gjernes and Måseide, 2015, 2020), dance (Kontos, 2004), therapeutic activities (Kovach and Magliocco, 1998), and cooking or baking (Hydén, 2014; Majlesi and Ekström, 2016).

For people living with late-stage dementia to perform actions, support from someone else is needed. As a result, many activities that previously used to be performed individually and independently, now turn into joint activities performed by the person with dementia together with a supporting other. The support must be based on the use of bodily actions and their coordination, as the possibilities of using spoken language is limited. Verbal directives describing actions—for example, “raise yourself from the chair by holding on to the walker, and then . . ..”—cannot be used as a prompt to make actions as persons living with late-stage dementia may have lost the ability to comprehend verbal directives or may not be able to perform them on their own. Hence, as the carer works together (collaborate) with the person living with late-stage dementia to perform actions and activities, there must be a shift from spoken language as the prime communicative resource to the use of other communicative resources for organizing the intercorporeal collaboration to perform actions.

In this article, we will show how it is possible to produce *concerted bodily actions* by reconfiguring how routine activities and actions are organized and performed in situ, so that the person living with dementia can participate in those activities. The reconfiguration of the context in situ requires, and is the outcome of, the systematic modification of the embodied conducts of the participants and their use of artifacts in the surrounding environment. The modification of the context of the production of actions makes it possible for the person with dementia to join in the activity and perform actions, for example, by following the carer’s use of her own body when she indicates and demonstrate how a conduct should be performed (cf. Goodwin, 2000). More specifically, we have identified three specific practices as central to the reconfiguration of the organization of the routine activities, all based on the local contingencies of the unfolding actions. All these practices are used to make it possible for the person with dementia to participate in tasks of daily living: (1) *staging* activities through organization and re-organization of body parts, as well as artifacts (rather than using verbal descriptions of activities); (2) decomposing (*parsing*) activities into smaller parts possible for the person with dementia to perform (rather than using verbal action descriptions); and (3) providing *embodied directions* and bodily *demonstrations* of actions (rather than using verbal directives). These three practices are mundane and used in many settings (not the least in learning situations) and are thus probably familiar to most participants. In the context of caregiving for people with late-stage dementia, these practices also involve a change of modalities: from using verbal language to visual depiction and bodily demonstration.

## Staging

Most activities, in particular activities involving bodily actions, pre-suppose a *staging* of the activity. Doing sports most often needs a special space, carpentry, the availability of tools, etc., as it does in other settings, for example, in medical interventions, etc. (see Heath, 2006; Hutchins, 1995; Raman, 2019). This staging is not only about providing space for action, but also a specific contexture through making available a variety of resources, tools and props, and organizing these in a way to enhance the performance of the activity (Goodwin, 2018; Hutchins, 1995; Kirsh, 1995; Nevile et al., 2014). The organization and re-organization of the “stage” of the activity is also a way to signal—communicate—what is about to take place or what is already taking place. This is especially important in joint activities when the participants need to coordinate their actions. How the working space is and how necessary tools for a particular activity are arranged are part of staging. Getting food from the refrigerator, placing spices behind the cutting board, and heating a frying pan, are all necessary organizational steps in cooking together. At the same time, it also has the function to indicate to participants what is going on and what the current state of the joint cooking activity is (Majlesi et al., 2019). Thus, staging the activity is a practice that involves not only the organization of artifacts, but also of participants who must take positions or be placed in the right positions to be ready for for example, medical examination (Heath, 2006), or washing (Twigg, 2000), or performing dance moves (Keevallik and Ekström, 2019) or martial art activities (Råman, 2019).

The organization of artifacts and participants (the “embodied participation framework,” see Goodwin, 2018) already *indicates* a set of instructions for the production of action (the arrangement of space and room is part of “semiotic fields” in the “contextual configuration” in Goodwin’s terminology, Goodwin, 2018). The indicating works by the location of artifacts or participants in certain places also makes it possible to “confer interpretations on the item that are placed there” (Clark, 2005: 515), highlighting them as figures against the ground. That is, it becomes possible for other participants in the activity to understand the placing of artifacts and persons as a “placing-for” (Clark, 2005) pointing to what in the situation is foregrounded and should be attended to, what the course of action looks like, and hence, what the next move might be. “Placing-for” as a way of indicating is thus a method for coordinating actions that participants perform, and showing also a temporal development, that is, what is coming about, and thus preparing for performing upcoming actions. Further, this also establishes figure-ground relations, making it easier for the participants to notice the indicated action as a figure to attend to in the context and in the temporal development of the activity.

## Parsing

In order to stage activities, it is necessary to understand the activity in a certain procedural order, and participants usually show each other their understanding of that order in terms of successively accomplishing various actions that compose the whole activity. As a person with late-stage dementia would usually have problems of understanding procedural tasks and steps in activities, to support them, the carer may thus need to decompose or parse the activity into more basic actions. Parsing in this context means a segmentation of “the fluid movements of skilled action,” so that the action is “‘seen’ as composed of strings of elements” (Byrne, 2003: 532). The elements identified “must meet one simple principle: each element should already be within the repertoire” (Byrne, 2003: 532) of knowledge and skill of the person living with late-stage dementia (i.e. whether they are able to understand and perform the action). When these smaller actions are performed, they can be stringed together into a fluid sequence of actions incrementally building the activity (cf. Ekström and Lindwall, 2014; Lindwall and Ekström, 2012; Råman, 2019).

Supporting eating activities for instance, implies that the carer can identify several smaller projects like rolling a person in a wheelchair to the lunch table, putting a plate, a spoon, and a glass in front of the person and a bib around the neck before the eating can begin (Ekström et al., 2022; Hydén et al., 2022). Thus, parsing an activity is not accomplished primarily as following an abstract cognitive scheme of instructions, but in terms of practical actions in which smaller tasks should be accomplished through various moves and set-ups. This may require for example, posing participants, or arranging artifacts and guiding participants’ behavior in a specific sequential order, such as moving the person, having them sit down, arranging plate and glass, etc.

Through parsing, it becomes possible for the carer to distinguish those smaller parts of the activity that needs to be performed by the carer, from the other parts that could be performed by the person living with late-stage dementia with various types of support from the carer (cf. Gjernes and Måseide, 2020).

## Directing and demonstrating

Parsing and staging practices as argued above are emergent properties of sequentially organized routine activities and they set the “scene” for activities by the organization of participants and artifacts. However, people living with late-stage dementia often need support to construe and carry out the required action that is expected of them. One of the basic ways of supporting people is by issuing directives, for instance giving someone instructions about what and how to do something, or to request that someone do something. Thus, a directive is a way to affect the behavior or actions of another person (Ervin-Tripp, 1976).

Directives can vary in force, from strong commands to indirect requests, and consequently they have different linguistic forms (Antaki and Kent, 2012; Goodwin and Cekaite, 2018). As pointed out by Grainger (1995), directives are ubiquitous in care contexts and are often used in carrying out activities like eating, washing, etc. (see also Antaki and Kent, 2012, and also Jansson and Plejert, 2014, and Heinemann, 2011 in dementia studies).

Directives based on spoken language often consist of a linguistic-descriptive account of the instruction or request (Ervin-Tripp, 1976). Directives can also be used either with minimized elements of spoken language or without the use of spoken language, for instance in cases of *embodied directives* (Goodwin and Cekaite, 2018; Majlesi et al., 2021). In the embodied directives, the linguistic description is substituted for a *bodily indication*, for instance making a request by pointing with the finger at something, making a demonstration through manipulating something with a finger or hand, or shifting attention by directing the gaze toward something. That is, the directive is performed as what Clark calls an indexical “pointing-to” (Clark, 2003, 2005). Central to understanding embodied directives—that is, for them to be understood as directives—is their sequential placement in the ongoing practical activity. That is, it is by being issued at a specific point in the ongoing activity that the bodily indication—perhaps together with some verbal utterances—may function as for instance an instruction of where to place a hand (as in sitting down) or when to open the mouth (as in eating). Placing the bodily indication at the right point in the activity thus makes it meaningful as a directive.

Different types of bodily indications can be combined with a demonstration of the action. For instance, demonstrating an action can be construed to model the action, that is, of showing both the action as such and how it is to be performed, something that is common in the sport context (cf. Råman, 2019). Demonstrating an action could also refer to depicting “physical scenes that people stage for others to use in imagining the scenes depicted” (Clark, 2016: 325). In the care context, this mean that the carer stages a demonstration of the action as an exemplary version of the intended action and a model to copy. The enacted actions “rely mainly on their visual, auditory, tactile, and proprioceptive knowledge of physical scenes and on their ability to use one scene in imagining another” (Clark, 2016: 324.). In other words, bodily experiences of the action are also included in the demonstration. It might for instance be the experience of standing in a certain position (Råman, 2019), holding and moving a crocheting needle (Lindwall and Ekström, 2012), or pronouncing a word in a certain way (Pekarek Doehler et al., 2021). The tactile and kinesthetic aspects of actions can thus be used for demonstrations (cf. Streeck, 2013, 2015).

In conclusion, what has been suggested is a conceptual framework that makes it possible to describe how routine activities and actions can be organized and re-organized in such a way that bodies, bodily parts like hands and fingers, as well as artifacts become salient and pivotal in the accomplishment of a next relevant action. They function as signals and cues that can be interpreted as being communicatively meaningful by the participants. By organizing and re-organizing the material ecology, it thus becomes possible to create, prepare and communicate the trajectory of ongoing activities, and by touching, pointing, and enacting actions, it is possible to coordinate and produce concerted bodily actions.

## Material and analytical procedures

The data material for this study consists of video recordings of an activity in which we analyze an event when Lisbeth, a woman in her eighties with late-stage dementia, is assisted into the kitchen and is engaged in scrubbing and peeling potatoes. The recordings were made during a 3-week period of ethnographic fieldwork in a nursing home where eight individuals all diagnosed with dementia were living. Two researchers were engaged in the data collection and spent whole or parts of their days in the nursing home during the data collection period taking part of the everyday activities there and interacting with both residents and care staff. As part of the field work, video recordings were made in the common areas of the nursing home—primarily the kitchen, the living room and hallway—using multiple stationary cameras.

Lisbeth was at the time of the recordings severely affected by her dementia, both motorically and even more so cognitively. She could move around the nursing home with support from care staff and a walker, and she could also perform basic tasks like eating with some guidance, but she often needed help to find her room and to know what to do and when. When talking to Lisbeth, it was often difficult to follow her narrative trajectory, and it was also hard to judge if she could understand what was said to her.

The recording of Lisbeth scrubbing potatoes was chosen as data for this study for several reasons. Firstly, Lisbeth’s remaining motoric functioning made it possible for her to engage in everyday activities in the nursing home. She could, for example, come along on walks in the garden or—as in the case presented in this study—participate in cooking and baking activities. However, her limited use of verbal communication made it necessary to use other means of communication in these activities. With our specific interest in embodied collaboration and interactive bodywork, Lisbeth was an illuminating case for further investigations as she, despite her very limited access to verbal communication, could participate in quite advanced collaborative activities with her remaining motoric skills. Secondly, the specific example with Lisbeth scrubbing potatoes was captured in its entirety and from multiple angles making the activity available for analysis in all its details, and therefore particularly suited as an example for this study.

Prior to the recordings, an ethical approval was obtained from the Regional Ethical Review Board, and recommendations provided by the Ethical Review of Research (CODEX) were strictly followed. To protect the identity of the participants, all names are pseudonyms, and all pictures used are anonymized illustrations.

The data is represented using transcripts following conventions from Conversation Analysis and Multimodal Interaction Analysis (Jefferson, 2004; Mondada, 2007). As there is not much talk used in the analyzed sequences, the transcripts mainly consist of descriptions of bodily actions and illustrative sketches showing the participants’ bodily positioning as well as the material surrounding.

Our analyses focus on how the participants engage in and collaboratively achieve the scrubbing of the potatoes using mainly non-verbal resources. We specifically focus on the sequential organization of the activity and how various communicative means are used and responded to, and how they contribute to the progressivity of the overall activity. The presentation of our analyses is guided by our three overarching concepts in focus for this study: parsing, staging and demonstrating.

## The episode

In total, the videotaped episode takes 7 minute 34 seconds. The episode has a clear beginning when Lisbeth and her carer, Lovisa, enter the kitchen area, and a clear ending when Lisbeth leaves the kitchen area assisted by another carer. The episode involves several sub-activities: (i) entering the kitchen, (ii) the preparation for the potato scrubbing, (iii) the actual scrubbing of potatoes, (iv) finishing scrubbing, (v) preparation for Lisbeth leaving the kitchen, and (vi) Lisbeth and her carer walking away from the kitchen. The sub-activities emerge from several smaller “local projects” (Clark, 1996) like moving pots, taking a potato in left hand while holding the scrubber in right hand, etc. In the episode a few sideplay sequences (Goffman, 1974) also take place involving various staff members talking about Lisbeth (whether she can stand on her own; or if she is tired) as well as other topics about the nursing home (we do not analyze these sequences here).

The analysis focuses on the first 2 minutes of the episode and involve sub-activities i-v above: how the activity of potato scrubbing is staged and parsed in Example 1a, and how the embodied directives are used for guiding and performing the scrubbing of the potatoes, and how a scrubbing sequence is accomplished in Example 1b and 1c.

## Staging and parsing the activity

Example 1a—a sequence with the length of 31 seconds—is about the start of the activity and how Lovisa—the carer—organizes her body position and also of Lisbeth as well as the position of the artifacts in order to start the main activity, the scrubbing of potatoes. This emergent local contingency is what we call staging of the potato scrubbing activity. Lovisa’s first task is getting Lisbeth into the kitchen area, guiding her to the sink, preparing the stage, including Lisbeth as part of it, for the potato scrubbing. The excerpt below starts with the carer Lovisa and Lisbeth entering the kitchen area from another room. Lisbeth has difficulties standing and walking; therefore, Lovisa holds her arm, supporting and guiding Lisbeth, while both are walking into the kitchen area (see Image 01; Line 1)).

**Example 1a.**
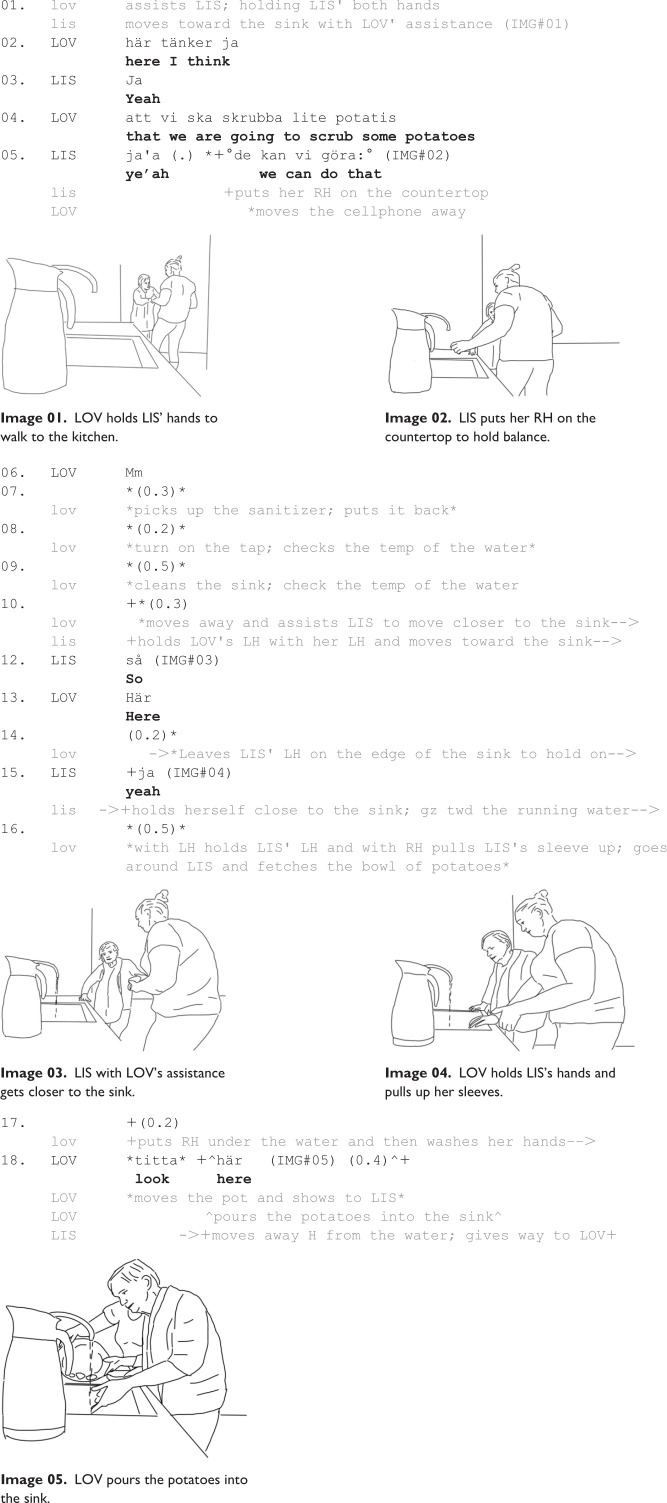
Participants: Lovisa (LOV; carer) and Lisbeth (LIS; pwd).

When Lovisa and Lisbeth are near the sink, Lovisa stops their joint movement, and at the same time introduces the upcoming activity as part of a planned sequence (using a mental predicate, “I think,” line 02), saying that they are going to be engaged in scrubbing potatoes (Line 04); something that Lisbeth confirms (Line 05). In first part of the whole activity, Lovisa moves Lisbeth toward the sink, while she at the same time verbally introduces the activity, something that Lisbeth apparently accepts, both verbally (by her uttering “yes” in several turns, e.g. lines 3, 5, and 15), and nonverbally, by moving along and following Lovisa’s verbal and embodied instructions.

### Staging

After entering the kitchen and coming closer to the sink, the next step that is, staging the activity, becomes prominent. Lovisa prepares the scene for the upcoming potato scrubbing activity. In order to be able to do this, Lovisa needs to stabilize Lisbeth to stand on her own, so that Lovisa can rearrange the place. Lovisa does this by helping Lisbeth to place her right hand on the countertop while she still holds Lisbeth’s left hand (Line 05; Image 02). In this way, Lovisa bodily guides Lisbeth to a certain location close to the sink to stand, freeing one of her hands to organize the materials on the countertop and the sink. Lisbeth’s standing by the sink also functions as a “placing-for”: Lisbeth is now part of a stage frame that indicate the possibility of a sink-related activity, that is scrubbing potatoes.

Lovisa rearranges the materials on the sink: she moves a cell phone and a sanitizer away, runs the water, and cleans the sink (Lines 5–9). Through these activities, Lovisa organizes and stages the sink in a preparation for the upcoming scrubbing activity. In other words, Lovisa’s organization of the sink functions as both practical and cognitive support for Lisbeth to engage in the upcoming activity. By removing irrelevant items, Lovisa foregrounds what needs to be attended to. Lovisa further makes room on the sink for the targeted activity of scrubbing potatoes, so that neither herself nor Lisbeth needs to be distracted by irrelevant items to the current activity and can more freely use the working space. Moreover, freeing the sink from unnecessary objects also provides cognitive support by making it easier to understand which objects that belong to the activity (i.e. to be salient) and should be used for its accomplishment. Placing irrelevant items in the background —in this case, the phone and sanitizer—diminishes the risk that these items will be in focus for Lisbeth’s attention, and she will potentially and more easily focus on those objects that are in the foreground of the activity (cf. Majlesi et al., 2019). The activity-relevant-artifacts are thus placed-for as salient “figures” for Lisbeth to notice also having the function to “scaffold” the activity (cf. Hydén, 2014).

Lovisa’s next project is to support and guide Lisbeth to adopt a standing working position (Lines 10–15; Image 03). Lisbeth’s participation in the joint activity is also apparent in her involvement in the coordinated movement and her verbal and embodied contribution to the accomplishment of the activity. For instance, when she got into a standing working position, she expressively displays that she in the position by saying “so” (line 12, so as a discourse marker signaling the transition between two activities, see also Majlesi et al., 2021), which is also rearranged by Lovisa: Lovisa (lines 13 through 15) guides Lisbeth’s right hand onto the countertop (Image 04), and thus Lisbeth can stand and hold herself by the sink (Line 15). A brief pause (0.5 second) marks the end of this phase. After making sure that Lisbeth is in a righ and safe standing position, Lovisa starts supporting Lisbeth washing her hands (Lines 17; Image 05). She also drains the potato pot into the sink (Image05) indicating that the scrubbing activity is about to begin. At the same time, she directs Lisbeth’s attention to the sink with the potatoes being poured into the sink and using a verbal directive (“look here”) which Lisbeth also follows by her own gaze (Lines 18).

Through embodied support, Lovisa has not only helped Lisbeth to move into the kitchen, but also staged both the sink for the activity, as well as guided Lisbeth into standing working position for scrubbing potatoes. She has also placed the activity-relevant items (here potatoes) in the cleared sink, making it easier for Lisbeth to notice them and treat them as relevant for the further activity by making sure that all necessary objects are within her reach. Further, it also facilitates for Lisbeth to understand the activity. This placing-for thus has a communicative function by indicating what is relevant in the situation while at the same time facilitating the performance of the activity by making sure all necessary objects are within Lisbeth’s reach.

### Parsing

Throughout staging, the activity is also parsed into smaller local projects, for instance “standing at the sink,” “washing the hands,” etc. These local projects all have a similar structure: (i) a start, (ii) the performance of the main action, and (iii) an ending (cf. Clark, 1996). The beginning and endings are marked either through verbal utterances or brief pauses, and there is generally some kind of acceptance of the projects, its beginning or end, by Lisbeth (cf. Bangerter and Clark, 2003). The starts are marked either by a small verbal utterance (Line 02; 05) or most often each is preceded by a small pause (Line 07, 08, 09, 10, 15, 16, 17, and 18). Similarly, pauses at the end of projects signal also their completion. Further, all the projects include some form of apparent verbal acceptance from Lisbeth (Line 03, 05, 11, 12, 15), probably indicating that she has noted what is going on and have no objections. Thus, Lovisa’s parsing of the preparations into smaller projects and Lisbeth’s acceptance of these projects, indicate that the preparation sequence is organized as a collaborative activity involving both participants. Although it is difficult to discern if Lisbeth’s produced acknowledgment tokens in conversation (e.g. “yeah”) are indicative of her mutual agreement in interaction, her minimal contributions to the conversation and her bodily collaboration are at this point treated by Lovisa to be in favor of the progressivity of the activity. In other words, the local projects are gradually proceeded without being challenged by Lisbeth, either verbally or bodily.

The performance of the local projects is therefore what incrementally drives the activity forward by adding a new project at a time. The local projects appear to be driven by how the ongoing activity sequentially develops in a hierarchical structure where certain tasks and actions are demonstrably considered as prerequisite of the next and small local projects are grouped together to build a larger one; for instance, the preparation of the activity and how the clearing the sink is followed by pouring the potatoes in the sink, the actual scrubbing of potatoes, and how making Lisbeth standing in a certain position occurs prior to her washing hands, etc. Lovisa is obviously oriented toward these larger activity units in her organization of the setting, staging and parsing the activity.

Finally, all activities take place in a shared visual space, making it possible for both participants to constantly see what is going on. Staging in terms of moving artifacts and moving Lisbeth by holding her arms, is not only a way to organize the activity but also has the function to communicate what is going on through the performance of the staging activity: Lovisa is showing Lisbeth visually and bodily where they are in the current activity and what the next step is.

## Directives and eliciting bodily enactment

In the continuation of the activity (Example 1b), as Lovisa has guided Lisbeth to stand by the sink in a working position, and potatoes are also within Lisbeth’s reach, the participants are ready to move to the next stage and scrub the potatoes.

**Example 1b.**
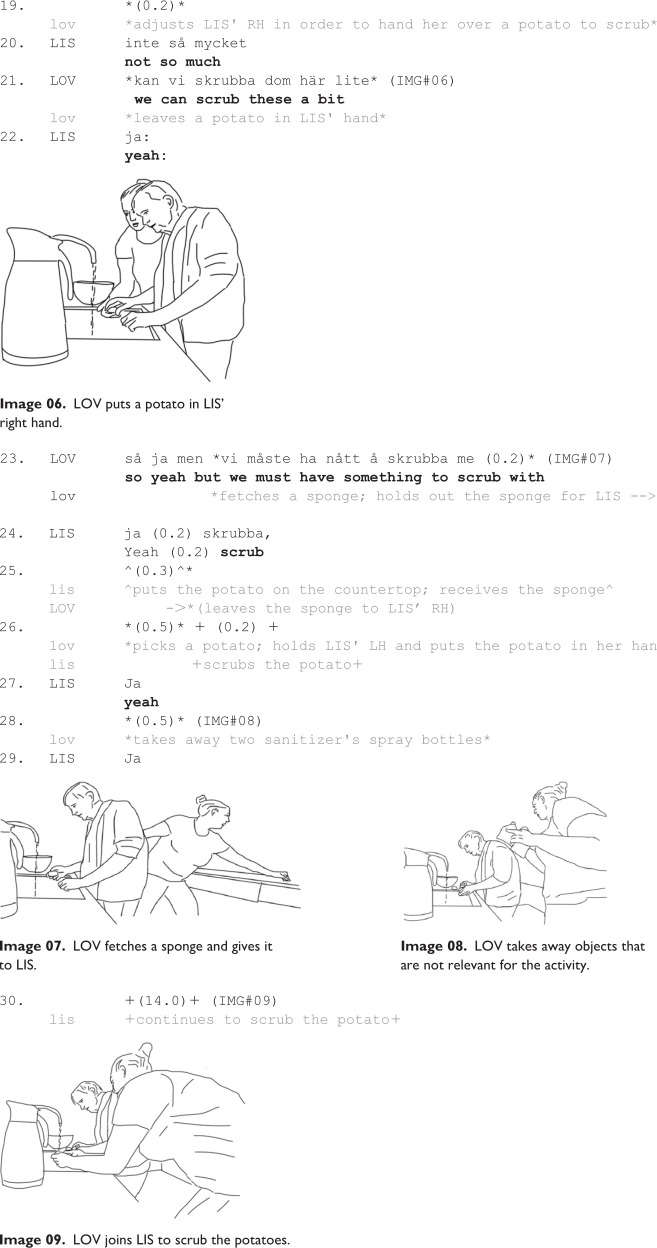


Lovisa starts the potato scrubbing by positioning Lisbeth’s hand, so that she can hold a potato (Line 19); that is, she again stages the activity by directing Lisbeth’s body. The reference of Lisbeth’s comment (“not so much,” line 20) is unclear, but Lovisa continues by saying “we can scrub these a bit,” (line 21), while she simultaneously puts a potato into Lisbeth’s right hand (Image 06). This functions as an embodied directive, and Lisbeth acknowledges this action by receiving the potato (Line 21) and providing an acknowledgment token “yeah:” (Line 22).

Lovisa then continues by looking for a scrubbing tool and finds a sponge on the countertop, on the other side of the kitchen. Lovisa fetches the sponge (Image 07), turns back and offers the sponge to Lisbeth. Lisbeth accepts the offer by putting down the potato that she had in her right hand and replaces it with the sponge (Lines 23–25). Then Lovisa puts a new potato into Lisbeth’s left hand (Line 26) and Lisbeth again says “yeah” at the end of this sequence (Line 27). It seems obvious that Lisbeth is more comfortable scrubbing with her right hand. So, when Lisbeth puts the potato down to receive the sponge, Lovisa immediately puts a new potato in Lisbeth’s left hand. Lovisa’s attending to the Lisbeth’s preference in holding the sponge with the right-hand and assisting her with putting a potato in her left-hand bodily guides Lisbeth to perform the activity. This rearrangement evidently helps Lisbeth to set off scrubbing. Lisbeth starts to scrub the potato and continues scrubbing this potato for about 75 seconds. In this sequence (Lines 21–26), Lovisa guides Lisbeth into starting to scrub the potatoes. Obviously, Lovisa’s guiding of Lisbeth’s hands (what to have in each hand) elicits Lisbeth’s scrubbing actions, and she continues on her own for quite a long time.

During the time Lisbeth is scrubbing potatoes, Lovisa takes away some spray bottles (Line 28; Image 08), and then starts herself to scrub potatoes, side by side with Lisbeth (Line 30; Image 09).

**Example 1c.**
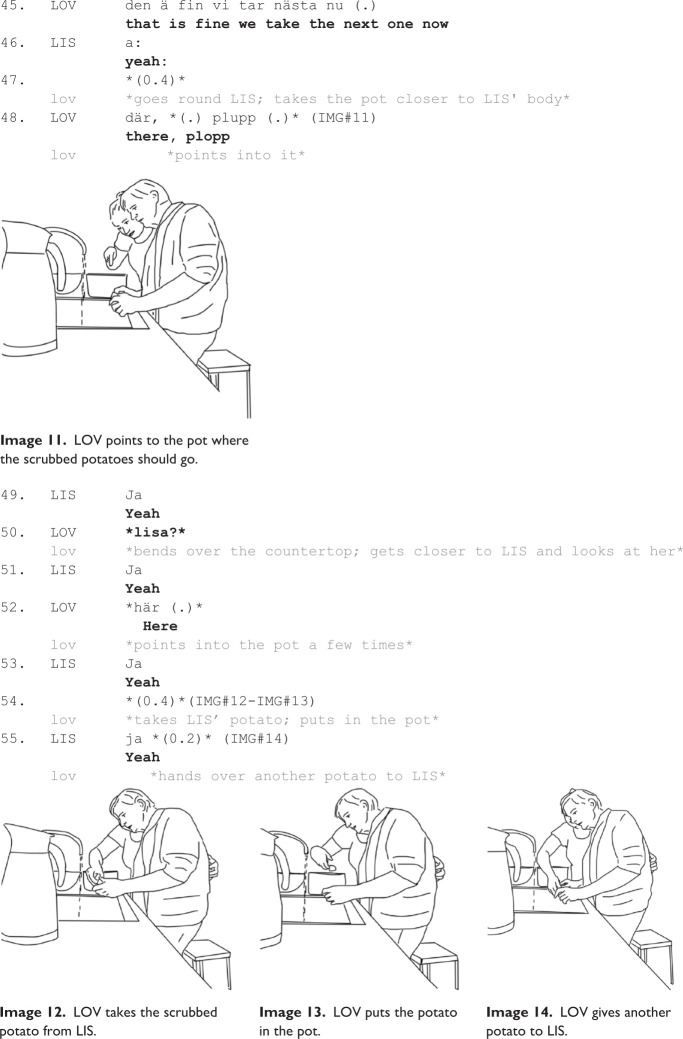


Holding on to the same potato and scrubbing it for 75 seconds shows that Lisbeth has problems knowing when the potato is adequately scrubbed, and also what to do next, that is, when and how to end the local project of scrubbing a potato. It is Lovisa who suggests ending the project by saying, “that is fine we take the next one now” (Line 45). Lisbeth says “yes” in response (line 46) but continues scrubbing the potato in her hand. Although Lisbeth verbally acknowledges understanding the directive that Lovisa provides for her to move on to the next potato, she does not practically comply with it, but continues scrubbing. Lovisa then goes around Lisbeth and pulls the pot on the countertop closer to the sink (Lines 47-8), points to the pot and says “there, plop” (Image 11). Lovisa is obviously addressing what she sees as a difficulty for Lisbeth, namely knowing what to do with the scrubbed potato and where to put it (Lines 48–53). However, such a demonstration with the help of hand gesture and the pot, showing visually what to do with the scrubbed potation does not work either. So, finally, Lovisa takes the potato from Lisbeth’s hand (Image 12) and puts the potato in the pot herself (Line 55; Image 13). Then Lovisa puts a new potato in Lisbeth’s hand (Image 14).

The sequences in Examples 1b and 1c are organized into projects, similar to Example 1a. The difference is that in Example 1a, most projects were preparations, staging the upcoming activity, while in Examples 1b and 1c, the projects are organized around parsing, directives and bodily guidance concerning how to perform scrubbing activities. Lovisa uses verbal directives, although there are few signs of Lisbeth bodily and practically complying with directives (besides her verbal acknowledgments timely produced after the directives). What seem to work is Lovisa’s embodied demonstrations and bodily guidance that provide Lisbeth with adequate resources to conduct the expected action. For instance, Lovisa puts the sponge and potato in Lisbeth’s hands, guides her to scrub—and Lisbeth then continue to scrub on her own. Finally, Lovisa demonstrates where to put the potato when it is scrubbed and how to pick up a new potato. Lovisa’s demonstrations thus function as an action guide for Lisbeth: what to do and how to do it (Lisbeth may not follow the instructions all the time, and sometimes requires help for performing the requested action, e.g. Lovisa takes the potato from Lisbeth and puts it in the pot when Lisbeth does not do as demonstration shows – i.e. Lisbeth does not perform what Lovisa shows by her pointing where the potato should be put, Image 11).

## Discussion and conclusions

The analysis of the potato scrubbing activity clearly shows that it is possible to organize and coordinate actions as intercorporeal collaboration. The verbal directives are few and it is unclear whether they are understood by Lisbeth. It is obvious that the activity is organized and performed primarily through the organization of bodies, hands and gazes as well as artifacts without the use of much talking. Lisbeth is guided by Lovisa from outside the kitchen area into the kitchen and placed and anchored by the sink. Things on the sink are moved away, receded to the background or prominently placed in the foreground of the scene. Lisbeth’s arms and hands are positioned for scrubbing, and Lisbeth is bodily guided to perform the scrubbing.

As our study highlights, the intercorporeal collaboration involves concerted bodily movements, also through the direct engagement of bodies with each other. This turns intercorporeal collaboration in caregiving activities into a form of bodywork. The term bodywork has been defined by Twigg (2000) as (paid) work “done on the bodies of others who thus become the objects of the worker’s labour” (p. 389). Heath (1986, 2006) stressed bodywork as an “ontological endeavour”: the construction and constitution of the body as “the site for clinical activity,” that is, as a medical object for the doctor to examine (1986: 187). Our analysis highlights the collaborative aspect of bodywork, namely what we can be called *interactive bodywork*: interacting *with* a body rather than working *on* a body and thus working with the *lived body* rather than a body as an object.

Interactive bodywork as a collaborative activity involves practices that are used to communicate and coordinate the participants’ conduct, for instance issuing verbal directives (using spoken language) that are supposed to be followed by the recipient. Other practices draw on the use joint visual attention like pointing-to or place-for something, while others also involve bodily conduct like modification of body positioning or conducting bodily performances (like demonstrations). Thus, different modalities are involved in these practices: linguistic, visual and bodily kinetics.

In bodywork based on collaborative activities, the participants are staffed different roles with different expertise and abilities (e.g. taking the role of a resident and a carer in the presented data). Thus, it is not surprising that participants adapt their practices to this asymmetric relation, and necessarily do so to accomplish an ongoing activity. We have also shown that reconfigurations of practices are part of contingencies in interaction. These reconfigurations and adjustments which take place over time through “trial and error,” and as it turns out, some methods are more feasible in order to, for instance, keep the person living with late-stage dementia as an active contributor in the activity, while others may not.

A general observation seems to be that in collaborative activities involving people living with late-stage dementia, there is a move from the use of linguistic practices toward the use of more visual and bodily kinetics, for instance bodily demonstrations (see Ingebrand et al., 2023, for learning strategies in joint activities involving people living with dementia). In this study, we identified three central practices: (i) staging and organizing the material surrounds including the participants themselves (ii) parsing the activity, and (iii) bodily guiding and supporting for the performance of actions. Through these practices, the person with dementia is included in the activity as they are positioned as an active partner.

We have also suggested a conceptual framework together with the analysis of the empirical example to unpack, understand and conceptualize mundane routine activities in caregiving settings. As an example, we demonstrated how scrubbing potatoes is performed by a person living with late-stage dementia and a care worker. Although comprising a great deal of daily care work, these everyday activities often tend to go unnoticed and thus remain unacknowledged (see also Buch, 2013; Harbers et al., 2002; Mol et al., 2010; Moser, 2010; Twigg, 2000; Twigg et al., 2011). It seems that these activities are seen as familiar, mundane and self-evident and thus of little interest. Contrary to that view, our conceptual framework and empirical analysis show that supporting people living with, for instance, late-stage dementia in the performance of everyday activities is quite complex in terms of organization and coordination and thus is worth studying. Our analysis does not only provide theoretical implications for understanding interaction with people with late-stage dementia as intercorporeal collaboration, but it also entails practical implications about the communicative, embodied sensitivity for building actions cooperatively with people in need of support.

The result of the analysis, overall, shows that intercorporeal collaboration is one of the most basic ways of supporting a person living with late-stage dementia, so it becomes possible for them to be an active participant in the activities, and thus to be recognized as an active and agentive person—although with bounded abilities.
